# Retrospective Observational Study of the Carbon Dioxide Insufflation Technique for Epicardial Access Ablation of Refractory Arrhythmias

**DOI:** 10.3390/jcm14248888

**Published:** 2025-12-16

**Authors:** Margarida G. Figueiredo, Bruno Valente, Leonor Magalhães, Guilherme Portugal, Hélder Santos, Ana Lousinha, Pedro Silva Cunha, Rui Cruz Ferreira, Mário Martins Oliveira

**Affiliations:** 1 Department of Cardiology, Hospital de Santa Marta, Unidade Local de Saúde São José, 1169-024 Lisboa, Portugal; 2 Arrhythmology, Pacing and Electrophysiology Unit, Department of Cardiology, Hospital de Santa Marta, Unidade Local de Saúde São José, 1169-024 Lisboa, Portugal; 3 Faculty of Medicine, University of Lisbon, 1649-004 Lisbon, Portugal; 4 Comprehensive Health Research Centre, 1150-082 Lisbon, Portugal

**Keywords:** catheter ablation, epicardial access, CO_2_ insufflation

## Abstract

**Background/Objectives**: Accessing intramural or epicardial arrhythmogenic substrates is limited in endocardial ablation, and in these circumstances, epicardial ablation may overcome these limitations. Because epicardial access may be associated with severe complications, intentional distal coronary vein exit and carbon dioxide (CO_2_) insufflation have emerged as techniques to facilitate safer access. **Methods**: We conducted a single-centre retrospective observational analysis of all patients who underwent epicardial ablation using the CO_2_ insufflation technique between September 2021 and October 2024. **Results**: Among 21 patients selected for the procedure, successful pericardial access was achieved in 19 (90.5%). The main indication for ablation was ventricular tachycardia (14 patients, 66.7%), and 16 patients (76.2%) had previously undergone endocardial ablation for the same arrhythmia. Most patients had non-ischemic cardiomyopathy (17 patients, 81.0%). Three intra-procedural complications occurred: one (5.3%) was access-related, and two (10.5%) were ablation-related. Early post-procedural complications occurred in three patients (15.8%). Acute procedural success was achieved in 16 of 17 patients (94.1%) who underwent ablation. In-hospital mortality occurred in three cases (15.8%), including one procedure-related death (5.3%). **Conclusions**: Intentional coronary vein exit and CO_2_ insufflation provide a safe and reproducible technique to obtain subxiphoid pericardial access for epicardial ablation.

## 1. Introduction

Endocardial catheter ablation has limitations, including the inability to access intramural or epicardial portions of arrhythmia circuits. Some of these difficulties can be overcome using an epicardial approach performed through the epicardial venous system or by percutaneous catheterisation of the pericardial space. Since its introduction in 1996 by Dr Sosa et al., percutaneous epicardial access is frequently required to target critical substrates and achieve successful ablation of a wide variety of cardiac arrhythmias in both patients with ischemic and non-ischemic cardiomyopathies [1,2,3]. This approach is often necessary either after endocardial ablation failure or as a first-line approach in specific substrates, such as ventricular tachycardias with epicardial circuits, Brugada syndrome with recurrent ventricular arrhythmias, complex cases of idiopathic ventricular tachycardias (VT), atrial tachycardias, including atrial fibrillation and atrial flutter (AFL), and accessory pathways (AP) that cannot be successfully targeted endocardially [4,5,6,7].

Nowadays, there are different techniques for epicardial access, from conventional pericardial access via a subxiphoid approach to the most recent intentional distal coronary vein exit followed by epicardial carbon dioxide (CO_2_) insufflation. Conventional pericardial access is associated with a risk of complications, including major adverse events, the most frequent of which is inadvertent right ventricular puncture (reported in 5–20% of cases), eventually leading to hemopericardium since it targets a “dry” pericardial space. Several approaches for epicardial access have been developed to minimise the risk of puncture-related complications. Among these, the novel technique of intentional distal coronary vein exit followed by epicardial CO_2_ insufflation has emerged as a means to clearly delineate cardiac borders [4,8,9,10].

The aim of this single-center retrospective analysis was to assess the efficacy and safety profile of epicardial ablation for various types of arrhythmias using the CO_2_ insufflation technique.

## 2. Materials and Methods

**Figure 1:**
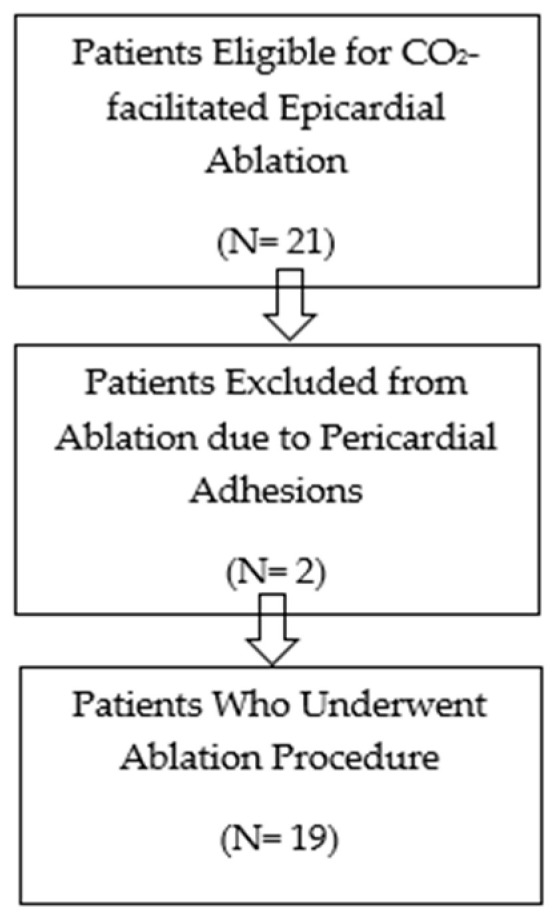
Flow diagram on participant selection.

This single-centre retrospective observational analysis includes all patients who met the criteria for epicardial mapping and ablation and, for that reason, underwent epicardial ablation using the CO_2_ insufflation technique over nearly 3 years, from September 2021 to October 2024. The study received ethics committee approval in January 2021. Although the study was originally designed prospectively, data were ultimately collected retrospectively from consecutive procedures performed between September 2021 and October 2024. The initial ethics approval covered the entire inclusion period, and no amendments were required. The inclusion criteria were considered when an epicardial arrhythmic substrate was suspected, based on prior failed endocardial ablation, imaging evidence of epicardial scar, and/or clinical conditions typically associated with predominant epicardial involvement. All patients provided written informed consent after being thoroughly informed about the nature of the procedure and its potential complications. The flow diagram for patient selection is shown in Figure 1.

Conscious sedation or general anaesthesia can be used for this procedure. However, general anaesthesia is typically preferred for epicardial procedures to improve patient comfort and minimise inadvertent movement during access, mapping, and ablation. In our analysis, the first seven procedures were performed under conscious sedation, whereas all the subsequent procedures were performed under general anaesthesia. While general anesthesia was the preferred anesthetic approach, resource limitations regarding its availability, combined with the electrophysiologist team’s experience in using deep sedation for other ablation procedures, resulted in some healthier patients being treated under deep sedation.

Antiarrhythmic medication was withdrawn at least 48 h before the procedure, except for amiodarone, which was maintained. Anticoagulation was adjusted to achieve an international normalised ratio of around 1.5, and direct oral anticoagulant agents were withheld 24 h before the procedure.

All epicardial ablations were performed percutaneously using CO_2_ insufflation. In some cases, concomitant endocardial ablation was performed during the same procedure. The type of ablation approach (epicardial only or endocardial plus epicardial) followed predefined clinical criteria, such as previous ablation attempts, type of myocardial disease, image exams including magnetic resonance imaging (MRI) and computed tomography (CT) scan. In all cases, an intrapericardial injection of methylprednisolone (1 mg/kg) was administered at the end of the procedure.

The first operator role was consistently shared between two electrophysiologists in all ablation procedures, ensuring procedural consistency.

**Figure 2:**
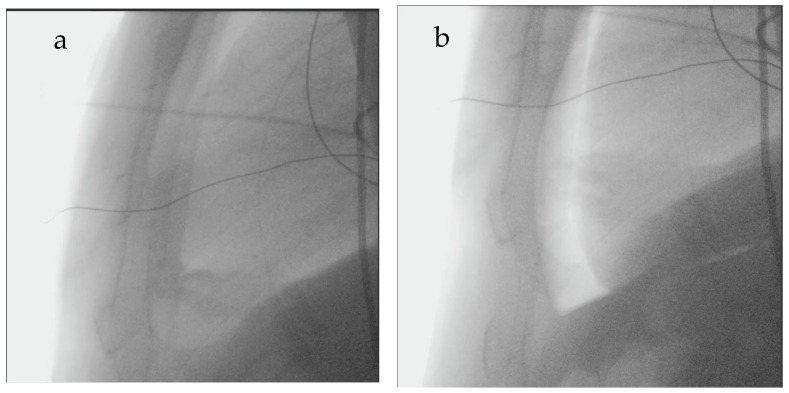
Epicardial CO_2_ insufflation technique. (**a**) Fluoroscopic image during initial epicardial access, showing minimal separation of the pericardial layers before CO_2_ insufflation. (**b**) Fluoroscopic image after controlled CO_2_ insufflation into the pericardial cavity. The radiolucent CO_2_ pocket creates a clear separation between the pericardium and the epicardial surface, improving visualization of the pericardial space.

Percutaneous epicardial access through intentional distal coronary vein exit with CO_2_ insufflation of the pericardial space and subxiphoid microneedle puncture was performed as previously described by Silberbauer et al. [10]. Briefly, using a femoral venous access, an ablation catheter was used to cannulate the coronary sinus (CS) and advance the Agilis introducer. After removal of the ablation catheter, fluoroscopic CS venography was performed, and a branch was then identified and subselected with a JR4 diagnostic coronary catheter and a 0.014-inch angioplasty guidewire. A high-tip-load wire was used to perforate the subselected target vessel, over which an ASAHI Caravel^®^ microcatheter (ASAHI INTECC Co., Ltd., 3-100 Akatsuki-cho, Seto, Aichi 489-0071, Japan) was advanced into the pericardial space. Contrast medium was injected before CO_2_ insufflation for two purposes: to verify accurate pericardial positioning and to rule out local adhesions that might restrict catheter manipulation, which are known to increase the risk of direct right ventricular perforation. CO_2_ was insufflated in serial boluses (typically using one or two 50 mL syringes, depending on heart size—larger hearts usually require more CO_2_ than smaller hearts) until the pericardium and cardiac border could be distinguished or the mean arterial blood pressure decreased by >10 mmHg—Figure 2. Pericardial puncture was then performed using a subxiphoid approach with a Tuohy needle under fluoroscopic guidance. Successful puncture of the pericardium was determined by advancing a guidewire through the needle into the pericardial space with confirmation of appropriate wire position by fluoroscopy and intracardiac echocardiography. A sheath was inserted into the pericardial space to facilitate epicardial drainage, for mapping, and ablation.

All procedures were performed under continuous peripheral invasive arterial pressure monitoring.

Successful epicardial access was defined as the ability to insert a sheath into the pericardial space following CO_2_ insufflation. All adverse events were documented and classified as ablation-caused or ablation-related. Pericardial bleeding was considered significant if >80 mL in the absence of a blood pressure drop. When the bleeding was associated with a decline > 20 mmHg in the systolic blood pressure, it was classified as cardiac tamponade [4,5].

Acute procedural success was defined as the absence of arrhythmia recurrence during the hospitalization period.

### Statistical Analysis

The normality of continuous variable distributions was assessed using the Shapiro–Wilk test, which is recommended for samples of fewer than 2000. Continuous variables with normal distribution were presented as mean ± SD; non-normal variables were reported as median (Q1–Q3). Categorical variables were expressed as percentages and numbers. All analyses were conducted using IBM SPSS software version 31.0.0.0 (IBM Corp., Armonk, NY, USA).

Missing values were handled using available-case (pairwise) analysis.

We acknowledge limitations regarding statistical inference due to the small sample size, namely inability to perform multivariable analysis or estimate predictors of access failure or complications.

## 3. Results

**Figure 3:**
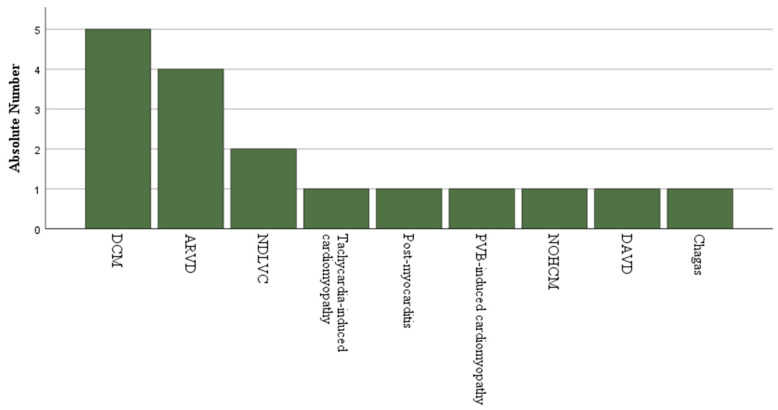
Underlying non-ischemic cardiomyopathy in patients undergoing epicardial ablation using CO_2_ insufflation. ARVD—Arrhythmogenic Right Ventricular Dysplasia; DCM—Dilated cardiomyopathy; NDLVC—Non-dilated left ventricle cardiomyopathy; NOHCM Non-obstructive hypertrophic cardiomyopathy; PVB—Premature ventricular beat.

Between September 2021 and October 2024, 21 patients underwent epicardial ablation using the CO_2_ insufflation technique. Baseline characteristics are summarised in Table 1. Most patients were male (71.4%), the median age was 64 years Interquartile range (IQR) (39–73), and the mean left ventricular ejection fraction (LVEF) was 45 ± 17%. A non-ischemic cardiomyopathy (NICM) was present in 81.0% of the cohort, and 81.0% had an implantable cardioverter-defibrillator. Figure 3 depicts the distribution of non-ischemic cardiomyopathy subtypes, whose classification was imaging-based.

Regarding prior procedures, 76.2% of patients had undergone at least one intervention targeting the same arrhythmia, most commonly an endocardial ablation (16 patients, 76.2%), with only one patient having undergone previous endocardial plus epicardial ablation (4.8%). Among the patients without any prior ablation or cardiac intervention, one had Chagas disease, one had a history of myocarditis, two had non-ischemic dilated cardiomyopathy, and one had arrhythmogenic right ventricular dysplasia (ARVD).

Additionally, two patients (9.5%) had previously undergone stellate ganglion ablation.

**Figure 4:**
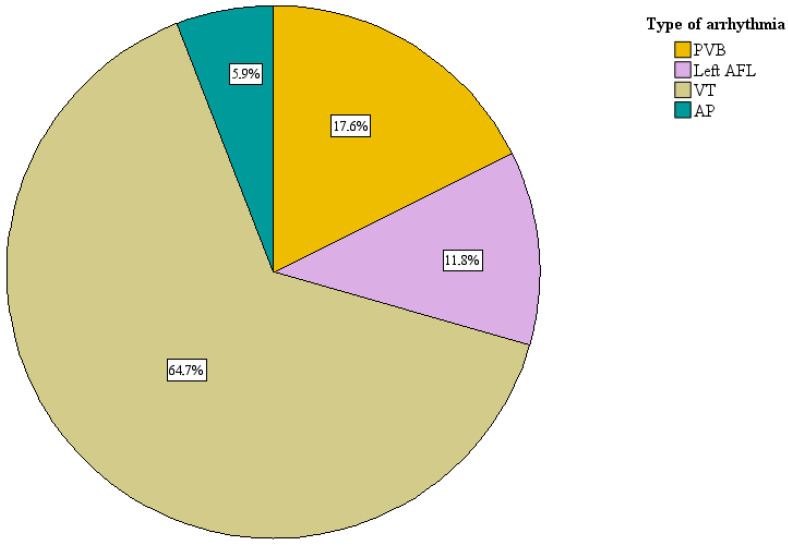
Distribution of arrhythmia types in patients with non-ischemic cardiomyopathy (NICM). AFL—Atrial flutter; AP—Accessory pathway; PVB—Premature ventricular beat; VT—Ventricular tachycardia.

The distribution of the type of arrhythmia in patients with NICM is described in Figure 4. Among the four patients with ischemic cardiomyopathy (ICM), the indication for ablation was monomorphic VT in the other three (75.0%) and premature ventricular beats (PVB) in one case (25.0%). Among patients with NICM, the indication for ablation was monomorphic VT in eleven patients (64.7%), PVB in three patients (17.6%), left AFL in two patients (11.8%) and AP in one case (5.9%).

Patients’ peri and intraprocedural characteristics are described in Table 2. Among the 14 cases of monomorphic ventricular tachycardia, eight arose from the left ventricle, three from the right ventricular outflow tract, two from the right free-wall, and one from the high septal region. All premature ventricular beats had a left ventricular origin: two from the anterolateral basal region, one from the left ventricular outflow tract, and one from the anterior basal region. The accessory pathway was localised to the posteroseptal region.

Of the 11 patients who had an urgent admission, the vast majority (10) presented with electrical storm—defined as a state of electrical instability, manifesting as three or more episodes of sustained VT within 24 h, separated by at least 5 min, and requiring intervention for termination-, and one presented with syncope. In three of the four patients with ICM, ablation was performed exclusively via an epicardial approach. Conversely, in 10 patients with NICM, a combined epicardial and endocardial ablation was performed, whereas the remaining patients underwent epicardial-only ablation. Electroanatomical mapping was performed using the CARTO^®^ 3 System Version 7 (Biosense Webster, Irvine, CA, USA) in 12 cases (57.1%) and the EnSite Precision™ Version 2.0.1. (NavX™) (Abbott, St. Paul, MN, USA) in 9 cases (42.9%). Mean radiofrequency time was 26.5 ± 14.2 min, while mean fluoroscopy time was 23.2 ± 10.4 min.

Table 3 presents the results for procedure-related complications, ablation success, and in-hospital mortality. Successful pericardial access facilitated by intentional CO_2_ insufflation via the coronary vein was achieved in 19 patients (90.5%). In the two cases in which pericardial access could not be obtained, both patients had a history of prior cardiac surgery (one with mitral valve replacement and one with coronary artery bypass grafting). The patient who had undergone mitral valve replacement presented with anomalous venous return, with the coronary sinus draining into the left atrium. In this case, an alternative approach with intentional pericardial exit via the right atrial appendage was attempted. However, in both patients, contrast injection revealed extensive pericardial adhesions, and CO_2_ insufflation was therefore not performed.

In patients in whom CO_2_ insufflation was used, epicardial access was achieved without any documented cases of inadvertent RV puncture or coronary artery injury.

Regarding intra-procedural complications in the 19 patients where successful epicardial access was achieved, only one was access-related, due to the coronary vein exit. This complication consisted of a significant haemorrhage of 330 mL; however, the patient remained hemodynamically stable, with no drop in blood pressure, and the bleeding resolved spontaneously during the procedure. The other two intra-procedural complications were related to the ablation procedure itself, namely: one cardiac arrest in pulseless VT, with recuperation of spontaneous circulation after 8 min of advanced life support, and one patient with a LV iatrogenic perforation after endocardial application of radiofrequency (50 W, force of contact 10 g). This last complication was the only one not related to a VT ablation, but rather to a PVB ablation; it was also the only ablation-related complication case in which a combined epicardial and endocardial approach was used and that required emergent cardiac surgery. In both the last case and the cardiac arrest case, the ablation procedure was not completed; therefore, these cases were not included in the procedure’s success rate analysis.

Concerning early post-procedural complications, all three were pericardial effusions, with only two requiring drainage—both occurring in patients who underwent combined epicardial and endocardial ablation. The other pericardial effusion occurred in a patient with epicardial-only access and was associated with pericarditis, which was successfully managed with non-steroidal anti-inflammatory drugs and colchicine.

Among the 17 patients who underwent ablation, 16 (94.1%) achieved acute success. The patient who experienced acute arrhythmia recurrence was a VT, with the recurring episode occurring during the index hospitalisation for the ablation procedure.

Median hospitalisation time was 2.0 (1.5–5.0) days, and in-hospital mortality occurred in 3 patients (15.8%). In one patient (5.3%), death was attributed to procedure-related complications, specifically an iatrogenic left ventricular perforation requiring emergent cardiac surgery. Among the remaining in-hospital deaths, one occurred in a patient in whom the ablation could not be performed due to intra-procedural cardiac arrest. This patient succumbed to recurrent ventricular tachycardia progressing to electrical storm and cardiogenic shock (arrhythmia-related). Another patient developed early post-procedural pericardial effusion requiring pericardial drainage. After clinical stabilisation, the patient was transferred to the referring hospital, where death occurred following a prolonged hospitalisation.

During a follow-up period of 8.0 (3.0–12.5) months, one patient had clinical pericarditis that was successfully treated with nonsteroidal anti-inflammatory drugs and colchicine. Two deaths occurred during this period, none of which were related to procedural complications.

## 4. Discussion

Percutaneous epicardial access has become an essential adjunctive tool in the catheter ablation of ventricular tachycardia and other arrhythmias. The conventional epicardial access targets a “dry” pericardial space, which presents technical challenges and a risk of complications. Significant pericardial bleeding is the most common access-related complication, with a reported incidence ranging from 3.7% to 10%. It is usually due to inadvertent right ventricular puncture due to the anterior location of this chamber and the narrow potential space between the right ventricle (RV) epicardium and the pericardium, which may be severe enough to require surgical repair [1,8]. In a study by Kumar et al., 1.7% of the patients undergoing this technique required operative intervention because of either RV perforation or coronary artery laceration, and procedure-related death occurred in 1% of patients [8]. Multiple methods have been developed over time to reduce epicardial access-related complications, including the “needle-in-needle” approach [8], “needle embedded with a real-time pressure/frequency monitoring” [11], “wire-guided” puncture [12] and, more recently, a concealed-needle blunt-tip device, which houses a fixated concealed needle, was designed to capture the parietal pericardium layer and retract it into the distal end of the device [13]. Although these techniques all sought to improve tools for pericardial puncture, none changed the very concept of puncturing a “dry” pericardium, and there was no significant reduction in major complications or significant bleeding with these approaches.

To improve the safety of epicardial access and prevent unintentional cardiac puncture, the epicardial CO_2_ insufflation technique was developed. The method consists of expanding the “dry” pericardial space with CO_2_ to increase the separation of the pericardium and epicardium, thereby not only increasing the size of the target zone but also enabling the clear identification of regions of adequate separation of pericardium and epicardium that can serve as appropriate targets for epicardial percutaneous needle access. This, in combination with an anterior microneedle puncture, also reduces the risk of infradiaphragmatic trauma [10,14].

Another important consideration is that the CO_2_ insufflation approach has significant value in early detection of adhesions and in potential avoidance of significant complications. In the presence of adhesions, the indication is to try a different location to determine whether the adhesions are localised or widespread. In the former scenario, advancement of the microcatheter is generally feasible after exiting at a different site; however, in the latter, this finding should preclude attempts to access the epicardium, thereby mitigating the risk of bleeding and other complications. In such cases, a decision should be made to either proceed with an endocardial-only ablation or to obtain a limited surgical window. For this reason, the use of this technique should be considered in specific patient populations, namely obese patients, those with chest wall deformities, or potentially those with prior surgery, in which pericardial access may be challenging, with increased risk of pericardial adhesions at the time of epicardial access [2,4,14].

The first thing to acknowledge in this study is that, as an inherent limitation of any retrospective study design, our analysis is susceptible to both selection bias and residual confounding.

We present various arrhythmias for which epicardial ablation may be considered. Moreover, the majority of our patients (76.2%) had previously undergone at least one ablation procedure.

Successful pericardial access facilitated by CO_2_ insufflation via intentional coronary vein exit was achieved in 19 patients (90.5%). This finding is consistent with previous studies employing CO_2_ insufflation for epicardial access, including the initial experience reported by Silberbauer et al. in 2017, which demonstrated a 92% success rate in twelve patients [10].

It should be noted, however, that in our study, intentional coronary vein exit (and one right atrial appendage exit) was achieved in 100% of cases, consistent with the results reported by Silberbauer et al. [10] (99%), and later by Juliá et al. [4] (100%). This indicates that the lower overall success rate for epicardial access was due to significant pericardial adhesions, which prevented attempts to achieve subxiphoid access. Notably, this technique offers a significant procedural advantage by avoiding unnecessary epicardial punctures that could otherwise increase the risk of complications [4,10,14,15].

Among access-related complications, only one event (5.3%) occurred in our study at the coronary vein exit. It consisted of significant bleeding without haemodynamic instability, which resolved spontaneously without intervention. This finding is also comparable with the rates of CO_2_ insufflation access-related complications found in the 2017 Silberbauer et al. study (8.3%) and later in the 2021 Epi-Co_2_ Registry by Juliá et al. (4.9%) [4,10].

One of the downsides of this technique is that it adds extra time to the procedure, since it requires an extra technical step—the coronary vein exit. However, once the coronary venous exit is achieved, the subsequent subxiphoid puncture is straightforward, and the overall procedure duration is expected to be similar to that of conventional epicardial access.

In our study, specific data on the time from coronary sinus cannulation to CO_2_ insufflation were unavailable. Nonetheless, the mean fluoroscopy time was 20.3 ± 10.2 min, which is significantly lower than that reported by Juliá et al., who documented a mean fluoroscopy time of 45 ± 23 min when using CO_2_ insufflation for epicardial access [4].

As for radiofrequency ablation time, the mean duration in our cohort was 26.9 ± 13.1 min, comparable to the results reported by Sacher et al. using conventional epicardial access (22 ± 16 min) [16].

Regarding intraprocedural complications, 2 cases (10.5%) were related to the ablation procedure, both necessitating premature termination of the intervention. One complication consisted of an iatrogenic left ventricular perforation associated with endocardial, rather than epicardial, ablation. This was the only event that required emergent cardiac surgery (5.3%). Pericardial effusion was the only early post-procedural complication observed in our study, occurring in 3 patients (15.8%), of whom 2 (10.5%) required pericardial drainage.

Regarding major complications associated with the conventional technique, Sacher et al. reported a rate of 7%, while Della Bella et al. reported a rate of 4.1% [16,17]. In the EpiCO_2_ Registry, ablation-related complications were present in 3% of patients [4]. A more recent study comparing conventional epicardial access to the CO_2_ insufflation technique demonstrated that major access-related complications were significantly reduced with the CO_2_ approach (0% vs. 7.5%, *p* = 0.02). Additionally, significant bleeding events related to the procedure were also lower in the CO_2_ group (5.5% vs. 17.5%, *p* = 0.02) [14].

When comparing our results with those of other studies, it should be noted that our cohort included both epicardial-only and combined epicardial–endocardial ablation procedures. Additionally, one ablation-related complication was attributable to endocardial ablation, reducing the intraprocedural complication rate to 5.3%, consistent with previous reports. Another important consideration is that, when implementing a novel technique, a higher complication rate may be observed in the initial cases due to the inherent learning curve associated with procedural experience.

In our study, among patients in whom ablation was successfully performed, acute arrhythmia ablation success was achieved in 94.1% of cases. This rate is superior to that reported in previous studies of epicardial ablation using conventional access techniques, such as the study by Santos et al., which documented an acute success rate of 87.5% [18]. Intra-hospital mortality occurred in three patients (15.8%), one of whom (5.3%) was related to the endocardial ablation procedure. Of these patients, two had previously undergone two endocardial ablation procedures, respectively. In contrast, the third patient had not undergone VT ablation but presented with an electrical storm characterised by incessant ventricular tachycardia refractory to all antiarrhythmic drugs and stellate ganglion block. These findings emphasise the need for alternative treatment strategies in patients with recurrent arrhythmia.

Uncertainty remains regarding which patients may benefit most from the epicardial approach. The described criteria aim to increase suspicion of epicardial arrhythmia origin [19,20].

In our patient series, most procedures were performed in individuals who had previously undergone endocardial ablation, except in specific cases in which, due to the high prevalence of epicardial scarring associated with these conditions, an epicardial approach was chosen as the initial approach.

Our study shows that the improved safety of CO_2_-mediated subxiphoid epicardial access allows consideration of epicardial ablation in cases that previously would not have been candidates for this approach [21].

Moreover, as demonstrated in prior studies, the success of epicardial ablation is closely linked to institutional and operator experience. We therefore anticipate that, with increased procedural experience and ongoing refinement of the technique, our outcomes will continue to improve over time.

### Study Limitations

This is an observational, single-centre, retrospective registry with a small sample of patients; for this reason, generalizability to other settings is limited. In addition, there is no comparison against a control group undergoing a conventional “dry” epicardial puncture, which renders definitive comparison of the safety and efficacy of the CO_2_ technique.

Furthermore, inherent to the retrospective design, our analysis is susceptible to potential selection bias and residual confounding. Data handling was managed using available-case (pairwise) analysis for missing values, which, while pragmatic, may affect the robustness of certain findings. We also acknowledge the lack of independent event adjudication and the potential for operator variability, given that procedures were performed by two primary operators.

Finally, we did not routinely monitor time from coronary sinus cannulation to subxiphoid epicardial access, and specific data regarding total procedure time were not available, which might have been essential and could be helpful for eventual comparison with other studies.

## 5. Conclusions

Our findings support the conclusion that intentional distal coronary vein exit followed by percutaneous subxiphoid anterior access using microneedle puncture after CO_2_ pericardial insufflation may be performed safely and reliably, even with relatively limited operator experience with this technique. However, given the small sample size and the highly selected nature of our patient cohort, the generalizability of these outcomes is limited, and we are unable to draw definitive conclusions regarding superiority over established techniques. Larger, well-designed studies are needed to confirm these findings in the future.

## Figures and Tables

**Table 1:** Patients’ baseline characteristics.

Number of Patients	21 (100%)
Male sex	15/21 (71.4%)
Median age (IQR), years	64 (39–73)
Underlying cardiomyopathy	
NICM	17/21 (81.0%)
ICM	4/21 (19.0%)
Mean LVEF, %	45 ± 17
LVEF < 50%	11/21 (52.4%)
Anticoagulation	12/21 (57.1%)
DOAC	10/21 (47.6%)
VKA	2/21 (9.5%)
Device therapy	17/21 (81.0%)
Type of device	
ICD	11/21 (52.4%)
CRT-D	6/21 (28.6%)
Type of prevention	
Primary prevention	8/21 (38.1%)
Secondary prevention	9/21 (42.9%)
Previous ablations/procedures	
Number	
0	5/21 (23.8%)
1	7/21 (33.3%)
2	9/21 (42.9%)
Type	
Endocardial only	16/21 (76.2%)
Endocardial + Epicardial	1/21 (4.8%)

CRT-D—Cardiac resynchronisation therapy with a defibrillator; DOAC—Direct oral anticoagulant; ICD—Implantable cardioverter defibrillator; ICM—Ischemic cardiomyopathy; IQR—Interquartile range; LVEF—Left ventricular ejection fraction; NICM—Non-ischemic cardiomyopathy; VKA—Vitamin K antagonist.

**Table 2:** Peri and intraprocedural characteristics.

Indication	
Monomorphic VT	14 (66.7%)
PVB	4 (19.0%)
Left AFL	2 (9.5%)
AP	1 (4.8%)
Type of admission	
Urgent	11 (52.4%)
Elective	10 (47.6%)
Presentation	
Electrical storm ^1^	10 (58.8%)
Diagnostic tests ^2^	7 (33.3%)
Syncope	3 (17.6%)
Appropriate ICD shocks	1 (4.8%)
Ablation access	
Combined epicardial + endocardial	11 (52.4%)
Epicardial only	10 (47.6%)
Mean radiofrequency time, minutes	26.9 ± 13.1
Mean fluoroscopy time, minutes	20.3 ± 10.2

AFL—Atrial flutter; AP—Accessory pathway; ICD—Implantable cardioverter defibrillator; PVB—Premature ventricular beat; VT—Ventricular tachycardia. ^1^ Electrical storm: defined as three or more episodes of sustained VT within 24 h, separated by at least 5 min, and requiring intervention for termination. ^2^ Diagnostic tests: included 24-h Holter monitoring or device-detected diagnosis.

**Table 3:** Procedure-related complications, acute procedure success and mortality during hospitalisation of the study population.

Successful Epicardial Access	19 (90.5%)
Ablation successfully completed	17 (89.5%)
Intra-procedural complications	3/19 (15.8%)
Access-related	1/19 (5.3%)
Ablation-related	2/19 (10.5%)
Cardiac arrest	1/19 (5.3%)
Iatrogenic ventricular perforation	1/19 (5.3%)
Early post-procedural complications	3/19 (15.8%)
Pericardial effusion	3/19 (15.8%)
Requiring drainage	2/19 (10.5%)
Acute success (when ablation completed)	16/17 (94.1%)
In-hospital mortality	3/19 (15.8%)
Procedure-related death	1/19 (5.3%)

VT—Ventricular tachycardia.

## Data Availability

The original contributions presented in this study are included in the article. Further inquiries can be directed to the corresponding author(s).

## Abbreviations

The following abbreviations are used in this manuscript:

AFL	Atrial flutter
AP	Accessory pathway
ARVD	Arrhythmogenic Right Ventricular Dysplasia
CO_2_	Carbon dioxide
CRT-D	Cardiac resynchronisation therapy with a defibrillator
CS	Coronary sinus
CT	Computed tomography
DCM	Dilated cardiomyopathy
DOAC	Direct oral anticoagulant
ICD	Implantable cardioverter defibrillator
ICM	Ischemic cardiomyopathy
IQR	Interquartile range
LV	Left ventricle
LVEF	Left ventricular ejection fraction
MRI	Magnetic resonance imaging
NDLVC	Non-dilated left ventricle cardiomyopathy
NICM	Non-ischemic cardiomyopathy
NOHCM	Non-obstructive hypertrophic cardiomyopathy
PVB	Premature ventricular beat
RV	Right ventricle
SD	Standard deviation
VKA	Vitamin K antagonist
VT	Ventricular tachycardia
